# Predicting response to immunotherapy in non-small cell lung cancer- from bench to bedside

**DOI:** 10.3389/fonc.2023.1225720

**Published:** 2023-11-15

**Authors:** Chris Montoya, Benjamin Spieler, Scott M. Welford, Deukwoo Kwon, Alan Dal Pra, Gilberto Lopes, Ivaylo B. Mihaylov

**Affiliations:** ^1^ Department of Radiation Oncology, Sylvester Comprehensive Cancer Center, Miller School of Medicine, Miami, FL, United States; ^2^ Division of Clinical and Translational Sciences, Department of Internal Medicine, University of Texas Health Science Center, Houston, TX, United States; ^3^ Department of Medical Oncology, Sylvester Comprehensive Cancer Center, Miller School of Medicine, Miami, FL, United States

**Keywords:** lung cancer, immunotherapy, radiotherapy, systemic response, local response, radiomics, murine, model

## Abstract

**Background:**

Immune checkpoint inhibitor (ICI) therapy is first-line treatment for many advanced non-small cell lung cancer (aNSCLC) patients. Predicting response could help guide selection of intensified or alternative anti-cancer regimens. We hypothesized that radiomics and laboratory variables predictive of ICI response in a murine model would also predict response in aNSCLC patients.

**Methods:**

Fifteen mice with lung carcinoma tumors implanted in bilateral flanks received ICI. Pre-ICI laboratory and computed tomography (CT) data were evaluated for association with systemic ICI response. Baseline clinical and CT data for 117 aNSCLC patients treated with nivolumab were correlated with overall survival (OS). Models for predicting treatment response were created and subjected to internal cross-validation, with the human model further tested on 42 aNSCLC patients who received pembrolizumab.

**Results:**

Models incorporating baseline NLR and identical radiomics (surface-to-mass ratio, average Gray, and 2D kurtosis) predicted ICI response in mice and OS in humans with AUCs of 0.91 and 0.75, respectively. The human model successfully sorted pembrolizumab patients by longer vs. shorter predicted OS (median 35 months vs. 6 months, p=0.026 by log-rank).

**Discussion:**

This study advances precision oncology by non-invasively classifying aNSCLC patients according to ICI response using pre-treatment data only. Interestingly, identical radiomics features and NLR correlated with outcomes in the preclinical study and with ICI response in 2 independent patient cohorts, suggesting translatability of the findings. Future directions include using a radiogenomic approach to optimize modeling of ICI response.

## 1 Introduction

Lung cancer remains the leading cause of cancer-related death both in the United States and globally (1). In the most common subtype, non-small cell lung cancer (NSCLC), the majority of patients present with aggressive, non-localized disease (2). For many patients with advanced NSCLC (aNSCLC) lacking driver mutations, the advent of immune-checkpoint inhibitor (ICI) therapy has improved overall survival (OS) and quality of life (QoL). While certain patients have a durable response to ICI with fewer side effects compared to chemotherapy, others experience clinically significant immunotherapy related adverse events (irAE). ICI monotherapy results in a lower burden of toxicity compared to chemoimmunotherapy and dual ICI regimens (3, 4), but with inferior objective response rates (ORR) and progression free survival (PFS) (3–6).

Biomarkers and models able to predict response to immunotherapy are helpful in guiding selection of intensified or alternative regimens, further personalizing patient care. Programmed death-ligand 1 (PD-L1) expression and the presence of microsatellite instability (MSI) are established predictive biomarkers (7, 8), tumor-infiltrating immune cells (IC) recently received FDA approval (8–11) and Tumor Mutational Burden (TMB) shows promise but remains under investigation (3, 12, 13). For these assays, the tissue samples needed for analysis can be imperfect or unavailable in various clinical scenarios, while cost and resource allocation can restrict access to the advanced laboratory techniques required.

Predictive models able to classify aNSCLC patients using readily available pre-ICI data alone could further optimize patient selection for treatment when tissue-derived biomarkers are unavailable or inconclusive. Baseline neutrophil-to-lymphocyte ratio (NLR) is obtained cheaply and has prognostic value for patients receiving ICI (14). Neutrophils are recruited by cytokines that also suppress anti-tumoral lymphocyte activity, and high ratios may reflect immunosuppressive conditions refractory to ICI response. Increases in NLR during ICI treatment, referred to as a positive **“**delta NLR**”** (ΔNLR), also may herald ICI resistance (15). A prognostic model, **“**ISEND**”**, was created for patients with aNSCLC treated with ICI monotherapy who had previously received first-line platinum chemotherapy (15). iSEND used clinical data (sex, ECOG performance status, NLR, and delta NLR) to sort patients into good, intermediate, and poor candidates for anti-PD-1 monotherapy. On independent validation, these groupings were predictive of overall survival (15). This promising effort required one NLR assay after the first cycle of ICI to generate ΔNLR.

There is ample pre-clinical and growing clinical evidence that radiotherapy (RT) as an adjunct to ICI can potentiate systemic disease response. This synergy is mainly due to RT**’**s ability to cause an immunogenic form of cell death that counteracts tumor immune escape mechanisms (16), and more speculatively to a phenomenon known as **“**abscopal response**”** (AR). The term **“**abscopal**”** (**‘**ab**’** - away from, **‘**scopus**’** - target) was coined in 1953 by R.H. Mole to refer to effects of ionizing radiation **“** *at a distance from the irradiated volume but within the same organism* **”** (17). In 2004, it was postulated for the first time that the immune system might be responsible for these **“**off-target**”** anti-tumor effects and subsequent preclinical work confirmed that the effects are in fact mediated by immunocytes (T-cells). It was therefore theorized that combining ICI and local RT could augment systemic response (18–20).. Trials in NSCLC have trailblazed the possibility of such effects (21–23). However, broad clinical evidence suggests that single site RT with ImT yields suboptimal treatment results in polymetastatic disease (24, 25). Comprehensive RT strategies targeting all measurable and even subclinical disease have been proposed in an effort to extend the role of RT to the polymetastatic setting (26, 27), often in conjunction with combination ImT when disease is refractory and/or tumor mutational burden (TMB) is low (28–30).

Quantitative image analytics, known as **“**radiomics,**”** can be used to characterize tumor clonal heterogeneity (31), among other aspects. Radiomic analysis assumes that patterns (known as **“**features**”**) below the threshold of visual detection are present within medical imaging and reflect underlying pathophysiology; converts medical imaging into mineable data and extracts clinically relevant features to improve cancer diagnosis, prognosis, prediction, and assessment of treatment response; and has augmented predictive models in diverse cancer types (32, 33) using conventional imaging studies such as computed tomography (CT) and magnetic resonance imaging (MRI). Advanced radiomics can quantify and analyze subregions within tumors which reflect differences in underlying tumor pathophysiology (31), such as lymphoid infiltration within highlighted areas (34). CT radiomics are of particular interest as these studies are commonly obtained for patients with aNSCLC, and CT texture features from pretreatment imaging have been incorporated in biomarker studies predicting for response to ICI (35, 36).

Previously, our group demonstrated that a preclinical murine model of aNSCLC incorporating pretreatment CT radiomics and laboratory data could be created to predict systemic response to immunotherapy after undergoing RT (37). In generating that model, radiomics features were identified that significantly correlated with systemic response. In the present study it was hypothesized that radiomics would augment development of a new predictive model able to classify treatment response of aNSCLC patients using pre-ICI data alone. Clinical outcomes between patients who had and had not previously undergone RT were compared to elucidate possible synergistic effect of ICI and RT. Respecting the multitude of biologic and radiographic differences between prior murine cohorts and human patients treated with ImT, texture feature analysis was carried out *de novo*, without consideration of prior significant findings. To evaluate the generalizability of this hypothesis, two distinct cohorts of aNSCLC patients treated with different anti-PD-1 monotherapies were interrogated retrospectively. The relationship between the findings in the murine model and in both human cohorts, and clinical implications of those findings, are presented and discussed below.

## 2 Materials and methods

### 2.1 Murine model

**Figure 1:**
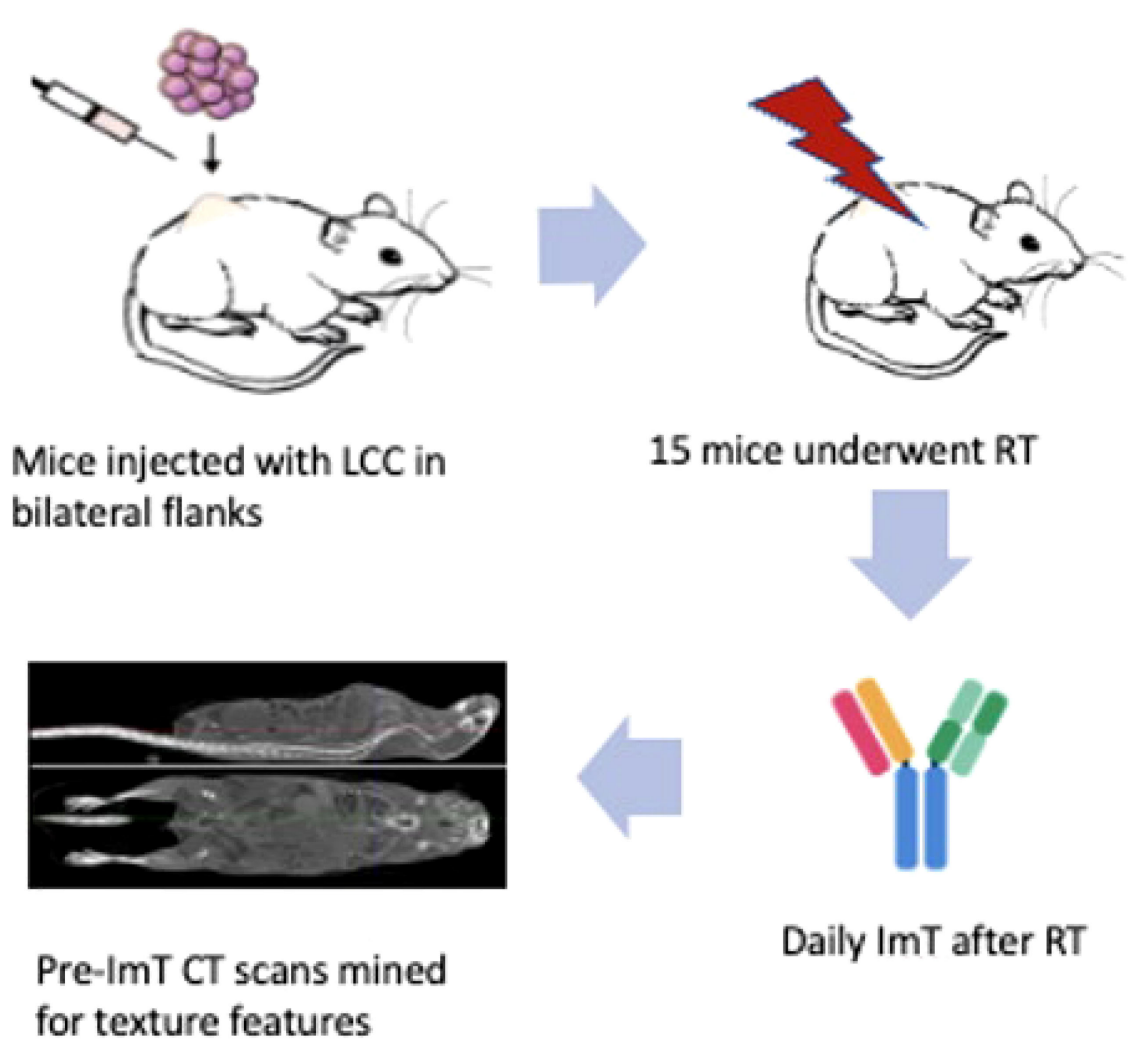
Workflow of the murine model. Nineteen C57BL/6 mice were injected subcutaneously in their *bilateral* flanks with 100 μL of Lewis Lung Carcinoma cell suspension. Four were designated as a control group receiving no treatment, and fifteen underwent RT and ICI. Baseline CT and blood tests were done on day 7. On day 8, mice in the experimental group were each irradiated. RT consisted of 8 Gy to the *right* flank only on 3 consecutive days. After each fraction of RT, treated mice received anti-mouse PD-1 immunotherapy. Both tumors were measured with digital calipers to assess both local (right flank tumor) and abscopal (left flank tumor) responses. No mice in the control group had a decrease in tumor size, while four mice in the experimental group had an abscopal response to RT and ICI. CT Scans of these four mice were mined for texture features associated with abscopal response for creation of a predictive model.

For the murine model, a cohort of nineteen C57BL/6 mice had 100 μL of Lewis Lung Carcinoma cell suspension injected subcutaneously into their bilateral flanks (animal protocol number 17-214-ad02 EDR, approved by IACUC on 1/27/2020). On day 7, baseline CT scans and blood counts were obtained. Four mice were used as a control group and fifteen underwent treatment. On day 8, treatment mice were irradiated using a RadSource 2000 X-Ray Irradiator cabinet under 2% isoflurane. All received 8 Gy to the *right* flank only on 3 consecutive days. After each fraction of radiotherapy (RT), treated mice received immunotherapy, consisting of intraperitoneal injections of 200 μg BioXcell anti-mouse PD-1 (CD 279). Both tumors were measured in all mice with digital calipers to assess both local (right flank tumor) and abscopal (left flank tumor) responses. Workflow is depicted in **Figure 1**. Further details of animal handling and the pertinent protocols can be found in an earlier publication (37).

Four mice were observed to experience a treatment response in both flanks, indicating enhanced systemic response. Left and right flank tumors on pretreatment CT scans were mined for texture features associated with tumor response. Each tumor had 92 CT radiomics features extracted. Features of interest consisted of geometric features, first-order histogram features, second-order joint probability features, and third-order joint probability features (previously described by our group) (38). All texture feature extraction was performed by in-house software, interfaced with the Pinnacle treatment planning system (Phillips Radiation Oncology Systems, Madison, WI).

### 2.2 Human cohorts

From an IRB-approved database (IRB number 20170427, approved on 6/19/2017), 117 consecutive aNSCLC patients treated with nivolumab monotherapy and 42 consecutive aNSCLC patients treated with pembrolizumab monotherapy between 2015 and 2018 were identified. The doses of nivolumab and pembrolizumab were 240 mg administered every 2 weeks and 200 mg administered every 3 weeks, respectively. Chart review collected baseline clinical characteristics (sex, smoking status, tumor burden, ECOG performance status, and prior RT) and laboratory data (WBC, ALC, ANC, EOS, PLAT, LDH, ALB). Various blood count tests (BCT) were generated, including neutrophil-to-lymphocyte ratio (NLR), platelet-to-lymphocyte ratio (PLR), neutrophil-to-monocyte ratio (NMR), and lymphocyte-to-monocyte ratio (LMR), in addition to prognostic nutritional index (PNI). PNI was calculated as 10*ALB (g/L) + 0.005*ALC (mm^3^). Positron emission tomography (PET)-CT scans immediately prior to initial ICI administration were transferred via Digital Imaging and Communication in Medicine (DICOM) files to a 3D treatment planning system (MIM v. 6.9.3; MIM Software Inc., Cleveland, OH) and fused to facilitate anatomic and target structure delineation. Radiomic analysis was performed with respect to the largest tumor on CT in accordance with prior radiomics studies in patients undergoing immunotherapy (39). In all patients, this also corresponded to highest standard uptake value (SUV) tumor on PET. For each patient, the largest tumor on CT was segmented using MIM software, with lung windowing used to define parenchymal margins and mediastinal windows used to define mediastinal and chest wall margins per established guidelines (40). Chart review was performed to establish clinical follow up and OS for all patients. As in the murine study above, 92 CT radiomics features were extracted for analysis.

### 2.3 Statistics

After compilation of laboratory data, clinical data (for human cohorts), and radiomics feature extraction, univariate and multivariate analyses (SPSS Statistics V.25 software package, IBM Corp., Armonk, NY, USA) were performed on all variables for dichotomized endpoints similar to prior work (37). The dichotomized endpoints were presence or absence of systemic response in murine model, and shorter or longer than median OS in the human cohort. The pairwise comparisons in the multivariate analyses were performed with Bonferroni adjustment for multiple comparisons. The imaging features, BCTs, and clinical variables (including pre-treatment variables used in iSEND model) with the highest statistical significance were selected for further modeling. All those selected variables were tested for correlation, with a Pearson correlation coefficient of 0.5 used for cut-off. If any two significant variables were correlated with a coefficient larger than 0.5, one of the variables was removed from the pool. The remaining uncorrelated variables were subjected to binary logistic regression, aiming to model the prediction of systemic response or OS. Both of the systemic response in animals and OS in humans were dichotomized. The systemic response was dichotomized in terms of present or absent, while the OS was dichotomized by median OS period. The binary logistic regression allowed to estimate areas under the receiver operating characteristic curve (AUCs) to assess the discriminatory accuracy in predicting systemic response in mice and overall survival in humans. SPSS ANOVA and SPSS Multivariate General Linear Model were used for these iterative analyses.

The murine and the human predictive models were subjected to internal k-fold cross-validation: 3-fold for murine and 5-fold for human. Each cohort was partitioned in k equal subunits (folds) and the model was trained on k-1 folds. Then the trained model was validated on the remaining 1 subgroup. The process was repeated k times with each subunit serving as training fold exactly once. In addition, the human model was externally validated on a separate patient cohort treated with pembrolizumab, a distinct anti-PD-1 ICI, to assess ability to discriminate based on OS. The results of the application of the binary logistic model to the pembrolizumab cohort were subjected to survival analyses (SPSS) where the survival rates were estimated using the Kaplan–Meier method and compared with log-rank tests.

## 3 Results

### 3.1 Murine predictive model

**Figure 2:**
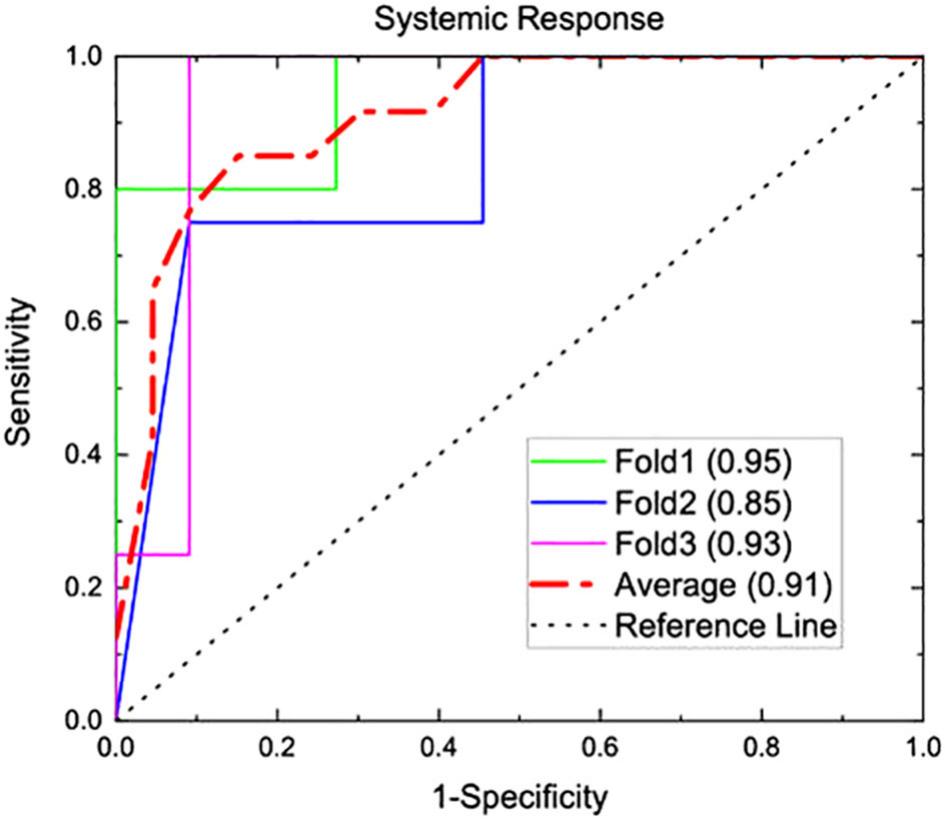
A predictive model was generated from the murine model of three texture features (surface-to-mass ratio, average Gray value, and 2D kurtosis), pre-treatment NLR, and pre-treatment II-1β. This underwent internal 3-fold cross validation for predicting systemic response, and AUC analysis for this prediction is shown above. A numerical average of these three folds yielded an AUC of 0.91.

Systemic response was observed in 4 of the 15 mice that underwent study treatment (37). From laboratory data, NLR and interleukin-1 beta (II-1β) were associated with systemic response (p<0.001 and 0.004 respectively). Three texture features (surface-to-mass ratio, average Gray value, and 2D kurtosis) trended toward association with systemic response on univariate (p=0.069, 0.013, 0.052) and multivariate (p=0.10, 0.05, 0.08) analyses. The selection of those features for downstream modeling was based on the univariate results. A logistic regression model incorporating these three texture features, NLR, and Il-1β for predicting systemic response yielded an AUC of 0.91 on three-fold internal cross validation. ROC analyses results from these validations are presented in **Figure 2**. Results of multivariate analyses for variables selected for predictive model are listed in **Table 1**.

**Table 1:** Results of multivariate analysis in murine cohort for association with systemic response for variables selected for predicted model, as described in Statistical Methods.

Texture Features	p-value	Hazard Ratio (95% CI)
Surface-to-mass ratio	0.10	1.141 (0.971-1.494)
Average Grey value	0.05	0.991 (0.975-1.007)
2D kurtosis	0.08	0.758 (0.544-1.058)
Laboratory Values		
Neutrophil-to-lymphocyte ratio	<0.001	0.122 (0.029-0.507)
Interleukin-1β	0.004	1.39 (0.963-2.008)

### 3.2 Human cohort characteristics

In the human nivolumab cohort, all patients had Stage IV disease, the vast majority had progressed after at least 1 prior line of systemic therapy, and adenocarcinoma was the most common histology (76%). The median age was 67 years, 65% were male, and only 17% were never smokers. Of the 13 patients for whom PD-L1 status was known, 10 were PD-L1 negative. Nivolumab had been FDA approved for the treatment of previously treated metastatic NSCLC regardless of PD-L1 status (8), and PD-L1 status was not routinely assessed at that time at our institution. The rest of the baseline characteristics are listed in **Table 2**. Median OS of the nivolumab cohort was 10.3 months and 2-year OS was 35%.

**Table 2:** Baseline clinical characteristics for human cohort receiving nivolumab.

Patient Race		
White	60	51.72%
Black	9	7.76%
Hispanic	42	36.21%
Asian	1	0.86%
Other	4	3.45%
Missing	1	0.85%
Total	117	100%
Patient Sex		
Male	65	55.56%
Female	52	44.44%
Total	117	100%
Body Mass Index		
Median	24.50	
Mean	24.77	
Age (years)		
Median	67	
Mean	66	
Smoking History		
Never	20	17.09%
<15 pack years	20	17.09%
15-30 pack years	17	14.53%
>30 pack years	60	51.28%
Total	117	100%
Pre-Treatment ECOG PS		
0	16	14.16%
1	20	17.70%
2	17	15.04%
3	60	53.10%
Missing	4	
Pathology		
Adenocarcinoma	74	63.25%
Squamous Cell Carcinoma	32	27.35%
Adenosquamous	1	0.85%
Bronchoalveolar	6	5.13%
Other^1^	4	3.42%
Total	117	100%
Number of Prior Systemic Therapies^2^		
0	6	5.17%
1	65	56.03%
2	29	25.00%
3	5	4.31%
4	11	9.48%
Missing	1	
Mean	1.57	
Median	1	
Relapsed or *de novo* Metastatic Disease		
Metachronous	40	34.19%
Synchronous	76	64.96%
PD-L1 Expression Status		
High (>50%)	2	15.38%
Low (1-49%)	1	7.69%
Negative (<1%)	10	76.92%
Missing	104	
Targetable Mutation Status		
KRAS	7	6.86%
EGFR	10	9.8%
ALK	3	2.94%
MET	2	1.96%
BRAF V600E	3	2.94%
None	77	75.49%
Missing	15	
Daily Steroid Use		
Taking	14	11.97%
Median Prednisone Equivalent Dose (mg)	11.68	
Blood Count Tests	Median	Mean
NLR	4.31	5.16
PLR	226.136	261.19
NMR	9.22	10.43
LMR	2.01	2.74
Primary Tumor Size (cc)		
Median	13.14	
Mean	52.98	
Nodal Disease at Time of ImT		
Yes	63	53.85%
No	54	46.15%
Anatomic M Stage		
**M1a**	49	41.88%
**M1b**	10	8.55%
**M1c**	58	49.57%
Overall Stage		
**IVA**	59	50.4%
**IVB**	58	49.6%
Prognostic Nutritional Index		
Median	43.3	
Mean	43.67	

^1^ Four patients found to have pathology other than NSCLC after chart review.

^2^ Excluding Avastin.

Among the 37 patients who had received thoracic RT prior to ICI, clinical intent, dose, and fractionation, as well as timing relative to ICI administration were heterogeneous. Overall, the presence of thoracic RT at any time prior to ICI showed no association with OS on univariate or multivariate analyses. Burden of disease was quantified using size of largest tumor on pre-ICI CT and number of lesions present on pre-ICI CT. There also was no association with either metric with OS.

### 3.3 Human predictive model

**Figure 3:**
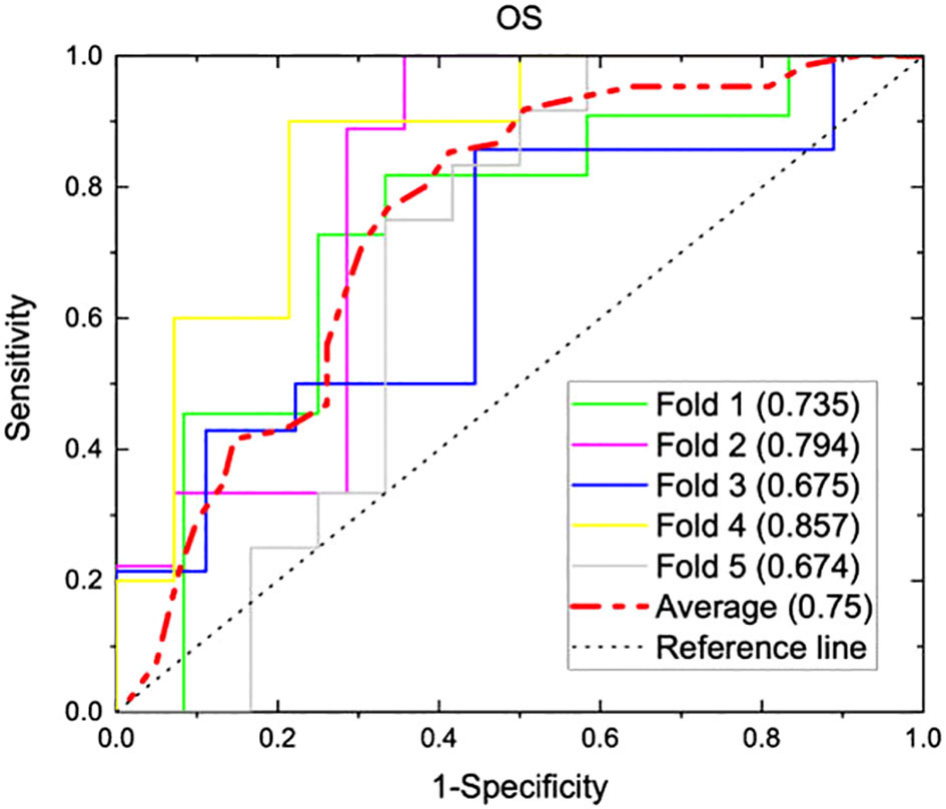
A predictive model incorporating three texture features (surface-to-mass ratio, average Gray value, and 2D kurtosis), ECOG performance status, pre-ICI NLR, and patient sex was generated from 117 aNSCLC patients treated with nivolumab. The model was internally validated with a five-fold cross validation, and AUC graphs for predicting OS were generated for each of the five folds. Individual AUC values are shown above, and the numerical average of the five folds was 0.75.

From radiomics analysis, three texture features correlated with OS on univariate and multivariate analysis: surface-to-mass ratio (p=0.032), average Gray value (p=0.02), and 2D kurtosis (p=0.018). Interestingly, those were the same 3 features which were associated with systemic response in the murine study. Furthermore, ECOG (p<0.001) and NLR (p=0.006) also correlated with OS. Female sex approached significance (p=0.08) on UVA for association with higher than median OS. A binary logistic regression model sorted patients into more likely or less likely to have longer than median OS, incorporating NLR, patient sex, ECOG performance status, the three texture features, age, histology, and number of prior therapies. Age, histology, and number of prior therapies were then excluded due to poor association, and a multivariate analysis was performed with only the remining variables. The cohort was then divided into five groups, and internal 5-fold cross-validation was performed. The average of the AUCs from each of the five folds (subgroups) yielded an AUC of 0.75 (**Figure 3**). Results of multivariate analyses for selected variables are tabulated in **Table 3**. Of note, those selected for inclusion in the predictive model include all pre-treatment factors from the iSEND model (sex, ECOG performance status, and pre-treatment NLR).

**Table 3:** Results of multivariate analysis in nivolumab cohort for association with overall survival.

Texture Features	p-value	Hazard Ratio (95% CI)
Surface-to-mass ratio	0.325	0.984 (0.952-1.017)
Average Grey value	0.594	0.998 (0.992-1.004)
2D kurtosis	0.040	0.996 (0.992-1.0)
Laboratory Values		
Neutrophil-to-lymphocyte ratio	0.117	1.046 (0.989-1.106)
Clinical Features		
ECOG Performance Status	<0.001	2.394 (1.663-3.447)
Female sex	0.057	0.688 (0.468-1.011)

Hazard ratios are reported for selected variables. Age, histology, and number of prior therapies were excluded and a second multivariable analysis was performed with the remaining variables. Texture features, NLR, ECOG, and sex were selected for inclusion in the predictive model, as described in Statistical Methods.

The coefficients from the five trained models were averaged to generate a representative average model. This average binary logistic regression model then was applied to a second, independent cohort of 42 patients with aNSCLC who received pembrolizumab monotherapy at the same institution. Like the nivolumab cohort, the pembrolizumab patients were all (100%) Stage IV but were relatively more fit (with 90.47% having ECOG PS of **≤**1) and less pre-treated (40.48% being treatment-naïve) than the nivolumab cohort. However, the two groups had very similar body mass index (BMI), primary tumor size, prevalence of nodal disease, relative proportions of anatomic M stage, BCTs, PNI, and proportion of synchronous versus metachronous metastatic disease. Pembrolizumab monotherapy was at the time of this study FDA-approved as first-line therapy for patients with EGFR/ALK wild-type aNSCLC and PD-L1 Tumor Proportion score (TPS) **≥**50% (8), and 24 of the 39 patients with PD-L1 status available had TPS **≥**50%. The rest of the baseline characteristics of the pembrolizumab cohort are listed in **Table 4**. The median OS of the nivolumab and pembrolizumab cohorts were 10.27 months and 14.27 months, respectively.

**Table 4:** Baseline clinical characteristics for human cohort receiving pembrolizumab.

Patient Race		
White	24	51.72%
Black	2	4.76%
Hispanic	15	35.71%
Asian	1	2.38%
Total	42	100%
Patient Sex		
Male	22	52.38%
Female	20	47.62%
Total	42	100%
Body Mass Index		
Median	25.27	
Mean	25.89	
Age (years)		
Median	71	
Mean	70.69	
Smoking History		
Never	3	7.14%
<15 pack years	7	16.67%
15-30 pack years	13	30.95%
>30 pack years	19	45.24%
Total	42	100%
Pre-Treatment ECOG PS		
0	6	14.29%
1	32	76.19%
2	3	7.14%
3	1	2.38%
Total	42	100%
Pathology		
Adenocarcinoma	34	80.95%
Squamous Cell Carcinoma	6	14.29%
Adenosquamous	1	2.38%
Bronchoalveolar	0	0%
NSCLC, NOS	1	2.38%
Total	42	100%
Number of Prior Systemic Therapies^1^		
0	17	40.48%
1	20	47.62%
2	4	9.52%
3	1	2.38%
4	0	0%
Total	42	100%
Mean	0.738	
Median	1	
Relapsed or *de novo* Metastatic Disease		
Metachronous	12	28.57%
Synchronous	30	71.43%
PD-L1 Expression Status		
High (>50%)	24	61.54%
Low (1-49%)	10	25.64%
Negative (<1%)	5	12.82%
Missing	3	
Targetable Mutation Status		
KRAS	2	6.90%
EGFR	4	13.79%
ALK	1	3.45%
MET	2	6.90%
BRAF V600E	1	3.45%
None	19	65.52%
Missing	13	
Daily Steroid Use		
Taking	7	16.67%
Median Prednisone Equivalent Dose (mg)	20	
Blood Count Tests	Median	Mean
NLR	5.16	6.46
PLR	220.97	248.30
NMR	9.39	12.71
LMR	2.01	2.30
Primary Tumor Size (cc)		
Median	11.10	
Mean	62.30	
Nodal Disease at Time of ImT		
Yes	31	73.81%
No	11	26.19%
Anatomic M Stage		
**M1a**	14	33.33%
**M1b**	5	11.90%
**M1c**	23	54.76%
Overall Stage		
**IVA**	19	45.2%
**IVB**	23	54.8%
Prognostic Nutritional Index		
Median	43.6	
Mean	44.27	

^1^ Excluding Avastin.

**Figure 4:**
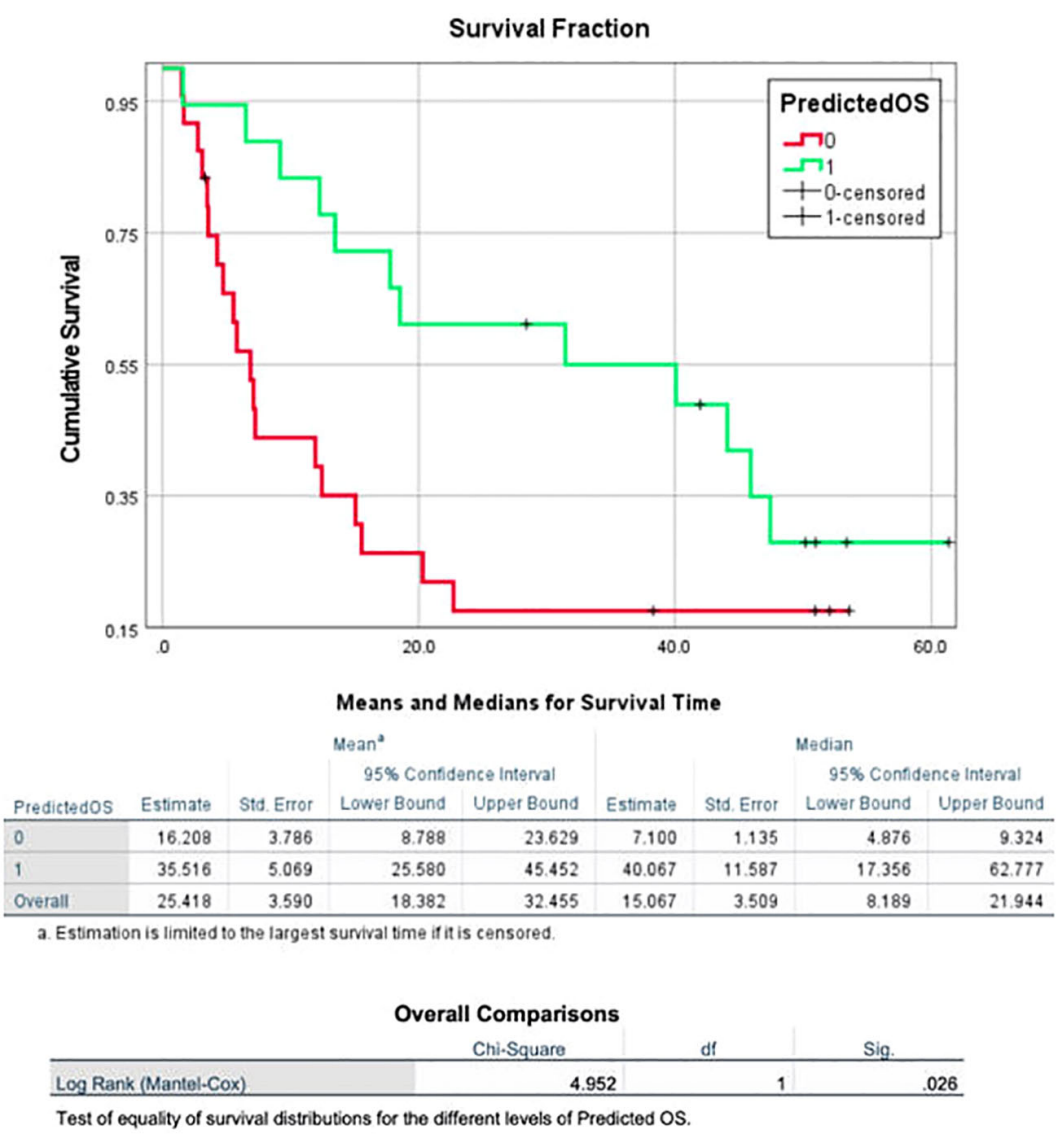
A model incorporating three texture features (surface-to-mass ratio, average Gray value, and 2D kurtosis), ECOG performance status, pre-ICI NLR, and patient sex for predicting OS in patients with aNSCLC receiving ICI, generated using a cohort of 117 aNSCLC patients treated with nivolumab, and then validated using a cohort of 42 aNSCLC patients treated with pembrolizumab. The cohort was dichotomized by the model based on predicted OS, and the two resulting groups had a statistically significant difference in observed OS.

**Figure 5:**
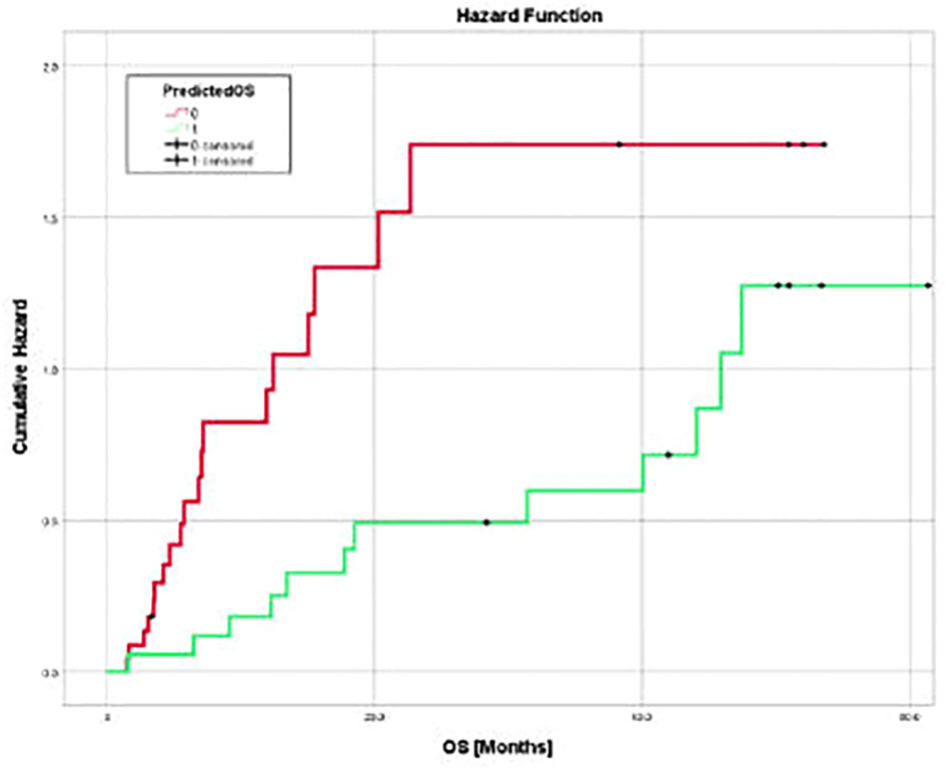
Cumulative hazard for death for two groups of pembrolizumab cohort sorted by predicted OS utilizing three texture features (surface-to-mass ratio, average Gray value, and 2D kurtosis), ECOG performance status, pre-ICI NLR, and patient sex. Cumulative hazard for patients with better predicted OS was significantly lower.

The model generated from the nivolumab cohort sorted patients in the pembrolizumab cohort into two groups according to predicted OS (**Figure 4**). The difference in observed median OS (35.75 months vs 6.98 months) of the two groups was statistically significant on log rank test (p=0.026). The hazard function of the two groups sorted by the model also showed significant difference in cumulative hazard (**Figure 5**). Statistical results are tabulated and summarized in **Table 5**. Combined with outcome data from the nivolumab cohort, these findings suggest that the regression model effectively discriminates patients who will derive clinical benefit from anti-PD-1 monotherapy.

**Table 5:** Tabulated overall survival values for two human cohorts, as well as pembrolizumab patient sorted into higher or lower predicted OS using predictive model.

Overall Survival of Pembrolizumab Patients (months)	Median
High Predicted OS	35.57
Low Predicted OS	6.98
Log-Rank	p = 0.026
Overall Survival of Human Cohorts (months)	Median
Nivolumab	10.27
Pembrolizumab	14.27
Log-Rank	p = 0.057

While OS differences were not significant on log-rank test between two cohorts overall, the difference between groups sorted by predictive model was statistically significant.

## 4 Discussion

For the majority of aNSCLC patients, ICI regimens improve treatment response compared with conventional chemotherapy (41–46). However, resistance to ICI is common due to the phenomenon of immune evasion, an emerging hallmark of cancer (47). Ionizing RT can prime cytotoxic immune response (16, 18–20), and has been shown to enhance ICI response in the metastatic setting (21–23). Improved predictive modeling of ICI response would help select candidates for treatment intensification or alternative therapies, and strategies to augment ICI response would improve clinical outcomes. Current biomarkers predictive of ICI response require invasive biopsy with the potential for high financial toxicity (48) limiting availability and access. Previously, the iSEND model (15) was developed to predict response to ICI monotherapy in aNSCLC patients using readily available clinical data, requiring delta-NLR determined by post-ICI assay. Subsequently, our group generated a model predictive of systemic response to ICI with RT in syngeneic mice inoculated with Lewis Lung Carcinoma, incorporating only pretreatment variables (radiomics and laboratory data) (37). Drawing on that preclinical experience, the present study attempted to improve predictive modeling of ICI-response by substituting quantitative imaging analytics of pretreatment CT for iSEND**’**s delta-NLR, while analyzing what relationship, if any, prior thoracic RT had with ICI response. While prior RT was not associated with clinical response, the dose, schedule, and timing of RT was heterogeneous. Prior investigations on abscopal response show dose-response to RT (21, 23), and it is difficult to interpret our results. The results of the present study (see Results section) support the use of radiomics as a tool to augment baseline clinical data in guiding precision cancer therapy for patients with aNSCLC.

Baseline NLR previously had demonstrated prognostic value for patients receiving ICI (14, 15), and its significance to both murine and human models in the present study was anticipated. An unexpected finding was the high translatability of tumor radiomics from syngeneic murine to human subjects, with identical texture features (average surface-to-mass ratio, average Gray value, and 2D kurtosis) emerging as predictive. Patients with known metastatic disease did not routinely undergo post-ICI tissue sampling and in this retrospective analysis extant samples were unavailable for molecular characterization. Interpretation of the tumor pathophysiology underlying the identified radiomics features therefore requires extrapolation from other sources.

Clonal evolution and epigenetic alterations drive heterogeneity in individual cancer cells within the same tumor. In NSCLC, genomic studies derived from tissue data have shown patients with higher measures of intratumoral heterogeneity are at increased risk of recurrence and death (49). Radiomics can characterize that heterogeneity in a non-invasive fashion (31). Advanced radiomics can identify subregions, sometimes referred to as **“**habitats**”**, within tumors that can be linked to differences in underlying tumor pathophysiology (31). The application of radiomics to tumor immune microenvironment (TIME) dynamics is a further development of the habitat concept. Our prior preclinical work demonstrated that radiomics of pretreatment MR and CT imaging can predict lymphoid and myeloid infiltration of tissue in regions of interest (34). In that study and the current one, the texture feature **“**CT average gray**”** emerged as predictive of treatment effect. This finding parallels a recent study showing CT gray level variance as predictive of tumor infiltrating lymphocyte (TIL) enrichment for patients with NSCLC treated with ICI (50). The FDA recently approved immunohistochemistry (IHC) assays of tumor infiltrating immune cells to predict ICI benefit in patients with NSCLC (51), and higher levels of CD8+ TILs has been associated with higher PFS for NSCLC patients treated with nivolumab (9). The correlation of predictive radiomics with lymphoid cell migration patterns suggests a potential role for quantitative imaging as an inexpensive, non-invasive alternative to IHC for prediction of ICI response. In addition, the ability of radiomics to localize lymphoid cell habitats within tumor, peritumoral stroma, and tumor draining lymphatics could define new therapeutic targets or avoidance structures for local or systemic interventions (52). Within the TIME, auto- and paracrine interactions reshape the extracellular matrix and contribute to local immunomodulation through recruitment and/or suppression of cytoxic T-cells or immunosuppressive regulatory T-cells (Tregs) (53). These changes in the local microenvironment promote tumor growth, angiogenesis (54), and regional lymphangiogenesis (55). In NSCLC patients, quantitative changes in Tregs and associated co-inhibitory factors within TIME are observed with progression of disease (53). Tumor neovascularization forms imperfect perfusion networks leading to regions of acute and chronic hypoxia, dynamic tumor proliferation rates, and clusters of variable cellular density. In our view, the radiomics features **“**average surface-to-mass ratio**”** and **“**2D kurtosis**”** most likely reflect tumor density and hypoxia status. 2D kurtosis has been shown to correlate with increased maximum SUV on FDG-PET, a correlate for increased tumor doubling time (56), and increased apparent diffusion coefficient on multiparametric MRI, a radiographic surrogate for density and hypoxia (57). Tumor hypoxia has long been associated with more aggressive disease and correlates with increased incidence of distant metastases in both pre-clinical and clinical data (58).

This study has significant limitations. Heterogeneity of systemic response to ICI in a syngeneic murine cohort with identical cell lines, identical inoculation sites, and identical interventions likely reflect variance of laboratory technique in the inoculation process despite all efforts to achieve uniformity. We believe that subtle changes in implantation of pseudometastases resulted in variable TIME dynamics and differential treatment effects, findings that were fortuitous and led to development of the present study. Patient data was compiled retrospectively, and confounding factors unaccounted for in our model may exist. While identical radiomics features may predict for clinical response in murine and human models, any similarity must be interpreted with caution. Intrinsic differences in the tumor immune microenvironment (TIME) between human NSCLC and subcutaneous murine pseudometastases influence the biologic processes predisposing either to ImT response (49). Heterogeneity of pre-ICI local and systemic therapies, profound anatomic and physiologic differences between real patients and any preclinical model, and lack of uniformity in CT imaging parameters can further confound radiomics findings and their interpretation.

**Figure 6:**
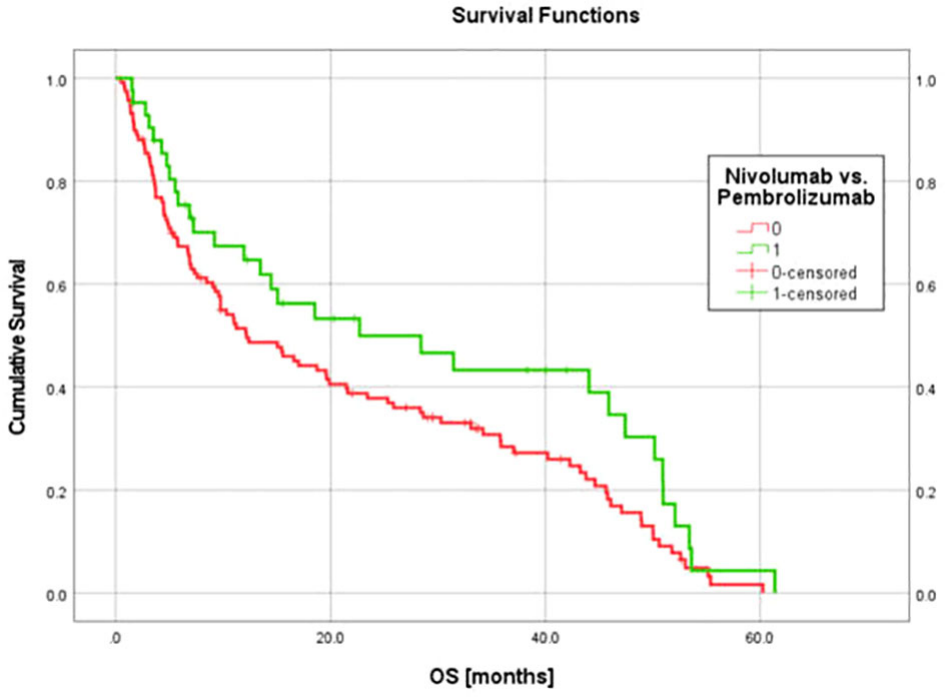
Kaplan-Maier OS curves for cohort of 117 nivolumab patients (used for model generation) and the cohort of 42 pembrolizumab patients (used for model validation). OS data for these patient groups were consistent with findings from external randomized prospective studies (41–43, 45, 46) and support the generalizability of the regression model derived and validated using these cohorts.

In the current study, the relatively large sample size and the simplicity of the biological correlates of the identified features may have reduced dependance on uniform imaging parameters to achieve reproducible results. CT measures relative (with respect to water) attenuation coefficient, a fairly uniform parameter across different machines compared to other imaging modalities such as MRI. Future protocols should consider standardization of CT scanner parameters to achieve a more harmonious radiomics signal. The original nivolumab model was validated with an independent group of patients who received pembrolizumab, however both patient cohorts were treated at the same institution, limiting generalizability of the results. Despite that limitation, the survival data for both cohorts in this study (**Figure 6**) are consistent with survival data observed in prospective trials of metastatic NSCLC patients receiving ICI monotherapy. In CheckMate 057, patients with advanced NSCLC having previously received chemotherapy and treated with nivolumab monotherapy had a median OS of 12.2 months for those with non-squamous cell carcinoma (42) and 9.2 months for those with squamous cell carcinoma (43). In KEYNOTE-001, metastatic NSCLC patients treated with pembrolizumab monotherapy had a median OS of 22.3 months in treatment-naïve patients, and a median OS of 10.5 months for those previously treated with chemotherapy (41). KEYNOTE-042 showed that for treatment naïve patients with aNSCLC and PD-L1 expression < 50% (33% of our dataset), median OS was 13 months (45). KEYNOTE-024 showed that for previously untreated patients with aNSCLC and **>**50% PD-L1 expression, median OS was 26 months (46), but high PD-L1 expression represented only 57% of our pembrolizumab dataset and the majority of that cohort were not treatment naïve.

Patients rarely underwent pre- and post-ICI tissue sampling and extant samples were unavailable for molecular characterization, making radiogenomic modeling impossible. Interpretation of tumor pathophysiology underlying statistically significant radiomics texture features therefore relies on considerable speculation.

Follow on studies will integrate tumor radiomics, clinical data, and molecular characterization to more fully elucidate ICI mechanisms of response in aNSCLC. Radiogenomic models will be optimized through concordance of TIME dynamics and genomics, longitudinally characterized by tissue biopsy, quantitative imaging, and liquid biopsy measures, and grounded in germline influences related to ethnicity and race. For patients with aNSCLC, multi-omics models are needed to guide strategic interventions that favorably adjust the mechanisms dictating clinical outcomes.

## 5 Conclusions

This study advances precision oncology by non-invasively classifying aNSCLC patients according to ICI response using pre-treatment data alone. Interestingly, identical; radiomics features and NLR correlated with outcomes in the preclinical study and with ICI response in 2 independent patient cohorts. Future directions include a radiogenomic approach designed to optimize modeling of ICI response and guide strategic interventions that favorably adjust the mechanisms dictating clinical outcomes.

## Data availability statement

The datasets presented in this study can be found in online repositories. The names of the repository/repositories and accession number(s) can be found below: https://doi.org/10.1371/journal.pone.0255923.s001.

## Ethics statement

This study was with the Declaration of Helsinki, and approved by the Institutional Review Board of University of Miami. The animal protocol number is 17-214-ad02 EDR, approved by the IACUC on 1/27/2020. The humans IRB number is 20170427, approved on 6/19/2017. Patient consent was waived for human cohort due to retrospective nature of study.

## Author contributions

Conceptualization, BS, IM, and SW; Methodology, BS, IM, and SW; Formal Analysis, BS, IM, and DK; Investigation, BS, IM, SW, and CM; Data Curation, BS, IM and DK; Writing – Original Draft Preparation, CM; Writing – Review & Editing, BS, IM, AP, SW, and GL; Visualization, BS, IM and CM; Mentorship, BS, IM, AP, SW and GL. All authors have read and agreed to the published version of the manuscript.
